# The Scent of Blood: A Driver of Human Behavior?

**DOI:** 10.1371/journal.pone.0137777

**Published:** 2015-09-23

**Authors:** James K. Moran, Daniel R. Dietrich, Thomas Elbert, Bettina M. Pause, Lisa Kübler, Roland Weierstall

**Affiliations:** 1 Department of Psychology, University of Konstanz, Konstanz, Baden-Württemberg, Germany; 2 Department of Human and Environmental Toxicology, University of Konstanz, Konstanz, Baden-Württemberg, Germany; 3 Biological Psychology and Social Psychology, Heinrich-Heine University, Düsseldorf, North Rhine-Westphalia, Germany; Knox College, UNITED STATES

## Abstract

The scent of blood is potentially one of the most fundamental and survival-relevant olfactory cues in humans. This experiment tests the first human parameters of perceptual threshold and emotional ratings in men and women of an artificially simulated smell of fresh blood in contact with the skin. We hypothesize that this scent of blood, with its association with injury, danger, death, and nutrition will be a critical cue activating fundamental motivational systems relating to either predatory approach behavior or prey-like withdrawal behavior, or both. The results show that perceptual thresholds are unimodally distributed for both sexes, with women being more sensitive. Furthermore, both women and men’s emotional responses to simulated blood scent divide strongly into positive and negative valence ratings, with negative ratings in women having a strong arousal component. For women, this split is related to the phase of their menstrual cycle and oral contraception (OC). Future research will investigate whether this split in both genders is context-dependent or trait-like.

## Introduction

### The scent of blood as a motivational driver

Human biological scents, including sweat, breath, breast milk and sexual effluvia appear to have a major influence upon human chemical communication, bonding and partner selection [1–3]. One bodily fluid that has been neglected in its possible importance thus far is the scent of blood. Surprising perhaps, because its presence in the environment is associated with fundamental survival, nutritional and reproductive factors, including cycles of female fertility, predatory behavior, as well as danger, fear, injury, prey, and death. Consequently, there are two theoretical motivational possibilities for the scent of blood: First, the appetitive approach stimulus e.g., associated with hunting prey; second the potential withdrawal danger signal, e.g. indicating that an area is dangerous. Most generally, approach is a response to a stimulus promoting survival, including sex, food, and nurture. Withdrawal is a response to stimuli representing threat, including, escape and self-defense [4]. A fundamental element of these motivational systems is emotion. In fact, emotions, though phenomenologically complex, seem to divide along motivational lines into approach and withdrawal related categories [4]. The qualities of the respective stimuli can be split into three dimensions: Valence (positive or negative), arousal (low or high) and dominance (low or high control) [5]. Therefore the first step in understanding the nature of blood scent is to gauge people’s emotional responses to it.

### Blood as approach vs. withdrawal stimulus

A recent study of predatory animals from [6], including wild dogs and tigers showed that they had a preference for blood over other scents. This was the case for both real blood, and an artificial scent made from an individual volatile component of blood. It can be argued that humans also have an analogous appetitive aggressive component in their behavior, considering the preeminent place of fighting, hunting and war in human society throughout its evolutionary history. Evidence from multiple disciplines, including ethology, anthropology, and psychology (reviewed in [7]), suggest that an ability to hunt and kill has reproductive and survival advantages, and moreover, that this selective pressure acts more strongly upon males than females [7,8]. Hunting is an arduous and dangerous task, involving extreme physical exertion, with prolonged arousal, and danger, as the prey defends itself in its final struggle. The ultimate reward of protein-rich food could be many days away. In these terms, those men who can enjoy immediate hunting-related stimuli, such as scent of blood and the cries of the stricken prey, would be the ones most likely to be successful in carrying it out [7]. Field studies in post-conflict lands reinforce the fundamental symbolic significance of blood in war. Soldiers have even reported craving the blood of the enemy, and finding the smell and taste of blood to be highly arousing [9]. Moreover, in a survey of combatants in war-stricken Eastern Democratic Republic of the Congo (DRC), 8% of ex-combatants reported having eaten human flesh, and 25% had observed others eating human flesh [9]. It is thus possible, through certain initiation rites, e.g., being forced by commanders to drink the blood of the slain enemy, that blood has become associated with reward and victory [10]. On the other hand, a more radical hypothesis would be that the smell of blood is an intrinsic and thus fundamental biological stimulus, evolutionarily prepared to signal approach towards prey. Therefore, in this model, blood would represent a fundamental approach motivational stimulus, as the scent of blood is one ubiquitous sensual element of killing. The English language features many metaphorical allusions to the love of blood ‘bloodlust;’ ‘getting a taste for blood;’ a first kill can be colloquially referred to as ‘first blood’ or ‘blood stripe.’ From the above observations, one can see these metaphors as potentially having literal roots, with blood as an appetitive approach stimulus.

In contrast, blood is often associated with withdrawal-related emotions, such as disgust, as evidenced by the preponderance of menstrual taboos in many different cultures [11], and the unique fainting reaction of blood phobia [12,13]. Brechbühl and colleagues [14] have found that stress related chemosignals in mammals and predator odors are processed in a specific olfactory subsystem known as the Grueneberg ganglion (GG). The GG is located in the rostrodorsal nasal vestibule. Its axons reach the caudal region of the main olfactory bulb, innervating the so-called necklace glomeruli [15,16]. The GG cells are considered to be part of the olfactory system, rather than the vomeronasal organ. Blood, in view of its association with danger, would be an obvious candidate for alarm specific scent processing in humans. Chemical communication of stress signals is based on sweat-related volatiles [17]. Sweat derived chemosignals produced through stressful situations can potentiate startle responses in humans [18], however this effect is moderated by trait social anxiety of the signal perceiver [19]. Indeed, rats performing a maze task avoided paths covered with spilled blood of a stressed rat, whereas this avoidance was not observed in paths with spilled blood of a non-stressed rat [20,21]. It was the stress related chemosignals rather than the scent of blood itself that influenced the behavior of the rats. The above observations thus demonstrate the importance of stress related chemosignals in sweat and blood. The influence of the scent of blood on human beings is as yet unknown: Do we react in an appetitive manner, as other predatory species have been shown to do [6], or do we process it primarily as a danger signal?

### Gender differences

Gender is an essential variable in assessing the response to the scent of blood. From a functional, behavioural point of view, women could have a differential likelihood of responding to the stimuli in an approach or withdrawal fashion. Evolutionary theory suggests that there was more selective pressure on men than women to develop hunting and killing of animals and rival conspecifics [7,8]. Though admittedly speculative, this finds empirical support in that women are more likely to identify with the victim than the perpetrator in the same imagined violent scenario [22]. Olfactory research examining responses to fear-induced sweat have shown that women are more sensitive to these anxiety cues [23,24]. In the most extreme case, women would interpret blood as an anxiety-related withdrawal signal, whereas men would interpret blood as an approach signal.

Another crucial variable relating to gender are the changes in response across the course of the menstrual cycle. Previous studies have shown that women’s response to odors or other social and sexual cues are strongly influenced by the phase of the menstrual cycle [25]. Pause and colleagues [26] have shown that information processing, indexed by time course of event-related potential (ERP) responses to scents, varied greatly across the menstrual cycle, with faster processing and greater verbalization seen in the ovulatory phase. Women’s varying preferences for more or less dominant men across the course of their cycle [27] are reflected in changes in their preference for androstenone, a characteristic male scent associated with testosterone levels [28]. Differences between the sexes, of course, allow many interpretations, foremost the fact that women, via menstruation, likely experience blood scent more often than men. Nevertheless any observable difference in the sexes would be a fundamental first step prior to further investigations.

### Chemical characteristics of the scent of blood

The scent of blood is a complex stimulus. It involves both the blood itself, as well as its reaction with surrounding substances. Volatiles within the blood can vary across species as well as from person to person. In fact, the variability of blood scent, and the difficulty of quantifying it via subjective assessment was a major theme in 19^th^ Century forensic medicine [29]. Nilsson and colleagues [6] approached this problem by using a compound found in a gas chromatograph analysis of blood itself, together with real blood, and testing this on predatory animals.

We take a different approach in operationalizing the scent of blood, by looking at its oxidization. In the case of a wound, blood reacts with fat lipids on the surface of the skin. An empirical test of the reaction of blood on the skin by Glindemann and colleagues [30] showed that a distinctive ‘metallic’ smell was produced, which was attributable to the oxidization of the hemoglobin’s iron molecules in the reaction with fat lipids in the skin. Gas chromatograph analysis of this reaction revealed the following volatile compounds: Hexanal, heptanal, octanal, nonanal, decanal, 1-octen-3-one [29]. The reaction could be suppressed by the addition of a blood-iron chelator, suggesting that the reaction related to the iron within the blood. A similar reaction, producing the same carbonyl hydrocarbon mixture was provoked by rubbing ferrous iron itself on the skin. Equating blood odor with ‘metallic’ qualities has further empirical justification from nutrition research. Im and colleagues [31] identify 1-octen-3-one as a volatile element of the smell of oxidized porcine liver, which is ‘metallic’, furthermore the ‘metallic’ taste of hemoglobin is cited as a problem in creating foodstuffs from slaughtered animal blood [32,33]. The mixture presented by [30] is based on analysis of only one individual, and one cannot rule out the possibility that volatiles may vary considerably from person to person [29]. The fact that both ferrous iron and blood of an individual rubbed on skin both analytically converge upon the same carbonyl hydrocarbon mixture argues against essential diffrences in blood, though maybe not skin. However, even if it were the case, universal perception of this particular scent is nevertheless highly likely. In the analogous case of androstadienone, only some people produce it, but it is recognized by most individuals [34] and might have signal function (discussed in [2]).

By artificially combining the components of blood scent [30], an artificial blood scent mixture was created. Using this simulated scent has the advantage of being non-variable and free of stress hormones. Since this has not been tested previously in humans, we examined the distribution of perceptual thresholds for this scent in an experimental population. For some ubiquitously present scents e.g., the synthetic musks in perfumes [35,36] and naturally occurring human semiochemical androstenone [37], there are qualitative differences in thresholds and quality of perception that are accounted for by genetic variations within a given population. For example, people who are markedly less sensitive to androstenone perceived it as more pleasant (associating it with sandalwood or flowers) than those who perceived it more acutely (associating it with sweat or urine) [34]. Consequently, it is important to analyze threshold simulated blood scent perception in order to detect potential differences within the experimental population.

### Hypotheses

The first task in assessing human motivational response to the simulated scent of blood in approach/withdrawal terms is to assess emotional response, i.e. is it positive or negative in valence, and is it high or low in arousal or dominance [38].

Threshold sensitivity to the simulated blood scent was also tested. Moreover, for these measures we also evaluated differences between men and women in these ratings, and whether women differ according to the stage of their fertility or contraception. Finally we tested whether there is any connection between evaluations of simulated blood scent and adaption of vegan/vegetarian lifestyle, to see if there is any relation between a person’s ethical standpoint on meat-eating and their physiological response to simulated blood scent.

## Materials and Method

### Participants

An experimental population of 89 people (44 women) was drawn from the student population at the University of Konstanz. Age had an a priori upper limit of 40 years of age, to avoid age effects on sensitivity [39]: Men (*M* = 25, *Range*: 20–40) and women (*M* = 24, *Range*: 20–39). The following medical conditions were used as exclusion criteria for participation: Any allergies; respiratory disorders; neurological disorders (including history of head injury; nervous system disease, epilepsy, concussion, migraine, Alzheimer’s disease, Parkinson’s disease, Korsakov Syndrome, Chorea-Huntington, Multiple Sclerosis); neurological surgery; nasal surgery (e.g., in the nasal septum); psychiatric disorders (depression, schizophrenia, eating disorders); hormonal disorders (e.g., thyroid problems); metabolic disorders (e.g., Diabetes); immune disorders (e.g., HIV); regular medication (e.g., blood pressure, antirheumatic, antihistamines, psychopharmaceutical, hormone treatment); acute infections of the upper respiratory tract.

Participants were told that for the day of testing they had to abstain from coffee, large meals, and smoking an hour before the test, and to refrain from drug or alcohol consumption in the preceding 24 hours before the test.

The testing procedure (vide infra) took around 1 hour, for which the participant was reimbursed with either 10 euros or 1 hour of experiment participation credit. Participants were debriefed at the conclusion of the experiment.

### Ethics Statement

The safety of the blood scent mixture (vide infra) was evaluated by an accredited toxicologist, who was part of the project (co-author DRD). The University of Konstanz Ethical Review Board approved the study and the experiment was carried out in accordance with the declaration of Helsinki. All participants gave written informed consent.

### Simulated blood scent solution

Using an artificial blood scent mixture based upon the analysis of [30] has many practical advantages. Firstly, we avoid participant exposure to real bodily fluids and other practical difficulties associated with using real blood (e.g., how to keep concentrations standardized; how to prevent coagulation without adding further substances). It also enables the creation of a precise dilution series for the evaluation of perceptual threshold, which would be unfeasible with an actual blood scent. Finally, in using synthetic blood scent, we eliminated other potentially confounding components, i.e. of sex and stress hormones in the blood.

### Control stimuli

In addition to determining the effects of the scent of blood, we offered three additional odors common in olfactory research with already established valence/arousal values, which could therefore serve as a reference for the blood scent and to validate the experiment [40–44] Dilutions of butyric acid (butanoic acid), an unpleasant smell found naturally occurring in dairy products, as well as in the gut and as a component of body odor, was used as a negatively valent comparison to the blood scent. 2-phenylethanol (an aromatic alcohol with pleasant floral odor) was also used as a positively valent scent in olfactory research. Finally we used pure water as a neutral reference point for ratings. We used only the butyric acid as a control for the threshold tests, as each threshold test took at least 15 minutes, and more than two threshold tests would be too demanding for participants. The floral odor and water controls for emotional ratings were introduced for the second batch of subjects only, i.e. for 24 male and 24 female participants.

### Threshold test

We used the threshold test created by Doty and colleagues [45] to determine the absolute perceptual threshold of the blood scent and butyric acid. First we created a series of dilutions of the blood scent mixture: The strongest concentration contained all 6 compounds mixed in EtOH and then diluted 1:20 in water to create the strongest concentration ‘1.’ The following 15 concentrations were progressively weaker, the size of the difference was in half-log 10 steps, thus; concentration ‘2’ was a 1:6.3 dilution of ‘1;’ and concentration ‘3’ was a 1:10 dilution of concentration ‘1.’ Concentration ‘15’ was a 1:10 000 000 dilution of concentration ‘1.’ A total of 7 perceptual staircase reversals were determined: 4 of which were ascending, and three descending. In each test, the participant was presented with two bottles to smell: One was a concentration of the simulated blood scent, the other, a neutral presentation of the solvent (mixture of H_2_O and/or EtOH). The participant was asked to rate which odor is stronger. The threshold is the mean of the last 4 of the total of 7 staircase reversals. This experimental design reflected the most reliable and widely used test for odor detection thresholds, and has been used for a large number of different odors [46]. The order of carrying out the threshold tests of blood scent dilutions and butyric acid solution was counterbalanced.

### Emotional rating of scents

Emotion ratings were assessed with the Self Assessment Manikin (SAM), whose rating scales assess three dimensions of emotion characterizing the approach/withdrawal response [38]: Valence, rated from -4 (negative), 0 (neutral), and 4 (positive); Arousal, rated from 0 (not aroused) to 9 (highly aroused); Dominance and -4 (submissive), 0 (neutral), to 4 (dominant)

Participants tested the scent once at the critical threshold, and once at an above threshold concentration. This was set at an above threshold concentration (solution ‘4,’ a 1:63 dilution of the baseline scent, see S1 Text for description of odor stimulus parameters including exact concentrations), consistent for all participants. Although one could keep the above threshold scent relative to individual thresholds, this would be less realistic from the point of view of ecological validity. We tested the relation between emotional ratings and perceptual threshold, to see if one influenced the other. The investigator cued them to give a number rating of valence, arousal and dominance by pointing to the respective pictorial series in front of them.

### Procedure

Odors were presented in brown 50ml bottles, with 30ml of the respective odor solution in each. The test room windows were opened between tests. Although this increased the potential influence of environmental odors, it was judged less of a potential problem than the accumulation of the very volatile experimental odors in the room with repeated testing. Concomitant personal assessment by experimenters did not detect the presence of any conspicuous external scents that could intrude on the experiment. To decrease potential remaining odors from the previous experimental round, the exterior of the bottles was cleaned with water and alcohol solution after each test. All surfaces were similarly treated. Experimenters wore gloves to avoid contamination of the bottles with other scents present on the hands.

When not in use on the day, the bottles were sealed with parafilm**®** and stored in a freezer (-18°C, except for the pure water) over night and on days where there was no testing.

The threshold test was carried out first for the blood scent and butyric acid dilutions. Then SAM ratings of the odor were made. After the odor threshold and SAM ratings, participants completed a short questionnaire on meat eating habits, asking whether the person was vegetarian or vegan (and some questions validating an indirect aggression scale for another study, not reported here). Finally, the female participants answered several questions on their menstrual cycle, namely if they took contraceptives, if so what type, the date of their last period and the length of their cycle. The fertility window was estimated using a fertility calculator, on the assumption that the female fertility window is approximately six days long [42]. While an individual hormone-based measurement would have been desirable to reduce noise, the fertility calculation has proven to be sufficiently robust in similar studies (e.g., [47,48]). Participants were also informally questioned afterwards regarding their impressions of the scent, e.g. what they thought it might be.

## Results

### Perceptual threshold

**Fig 1:**
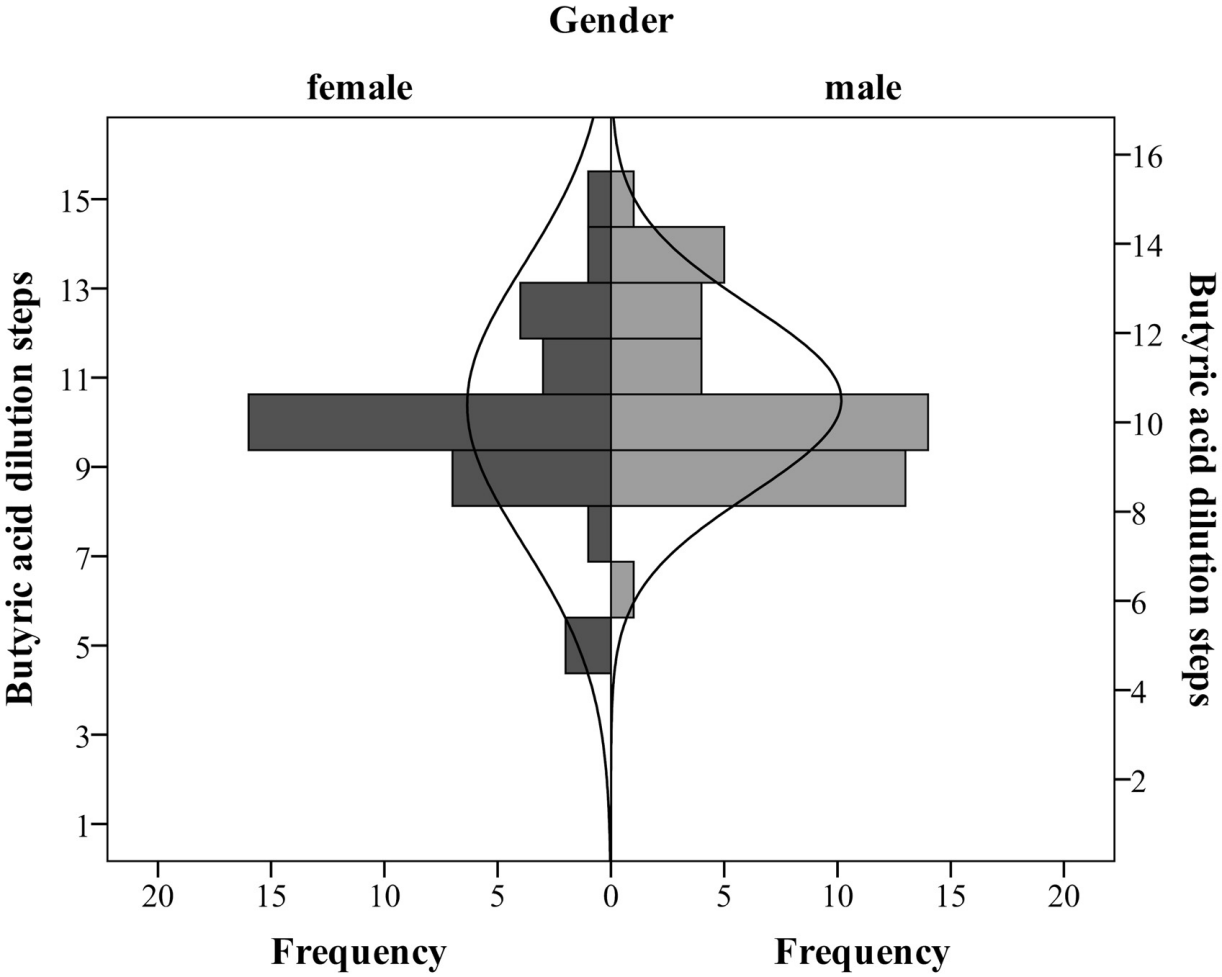
Butyric acid threshold. Distribution of butyric acid perceptual thresholds separated for gender. The solid line shows theoretical normality curve. Number 1 on the y-axis represents the original concentration, and each following represents a dilution of one half log 10 step, so higher values represent higher sensitivity.

**Fig 2:**
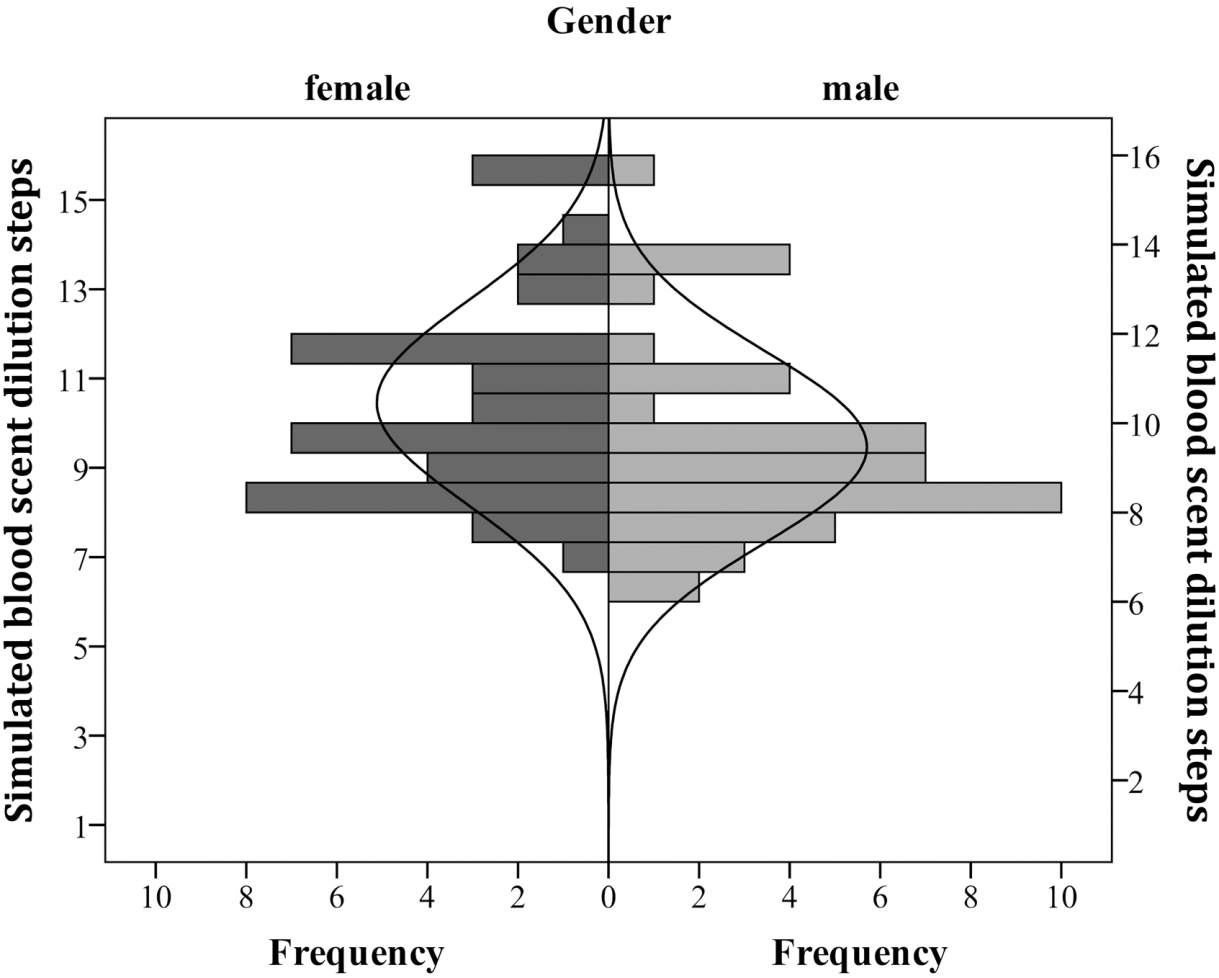
Simulated blood scent threshold. Distribution of simulated blood scent perceptual thresholds separated for gender. The solid line shows theoretical normality curve. Number 1 on the y-axis represents the original concentration, and each following represents a dilution of one half log 10 step, so higher values represent higher sensitivity.

Normality statistics, namely the Shapiro-Wilkinson test, showed significant deviation from normality for the perception of simulated blood scent (*W* = 0.921, *p* < .001) as well as for butyric acid (*W* = 0.928, *p* < .001). However this was most likely attributable to a significant negative skew for simulated blood scent, with *p* <0.001, and a significant kurtosis for butyric acid, with *p* <0.001, suggesting a heavy-tailed distribution. There do not appear to be two or more distinct populations showing differential sensitivities for the perception of simulated blood scent. Men and women did not differ in regard to the unimodality of distributions (see Fig 1, butyric acid and Fig 2 simulated blood scent), however a Mann-Whitney-U test revealed that women (*Mdn* = 10.25, [*Range*: 7.5 to 15.5]), were significantly more sensitive to simulated blood scent than men (*Mdn* = 8.75, [*Range*: 6.25 to 16], *U* = 696, *p* = 0.016, *r* = 0.26). This gender difference was not present for the butyric acid comparison (women: *Mdn* = 10.00, [*Range*: 0 to 16], men: *Mdn* = 9.75, [*Range*: 6.25 to 16], *U* = 816.5, *p* = 0.838, *r* = 0.02). There was no correlation between threshold perception and age (*Range* = 20–40 years) for either simulated blood scent (*r*_*s*_ = - 0.13, *p* = 0.242) or butyric acid threshold (*r*_*s*_ = - 0.10, *p* = 0.374).

### Emotion ratings

**Fig 3:**
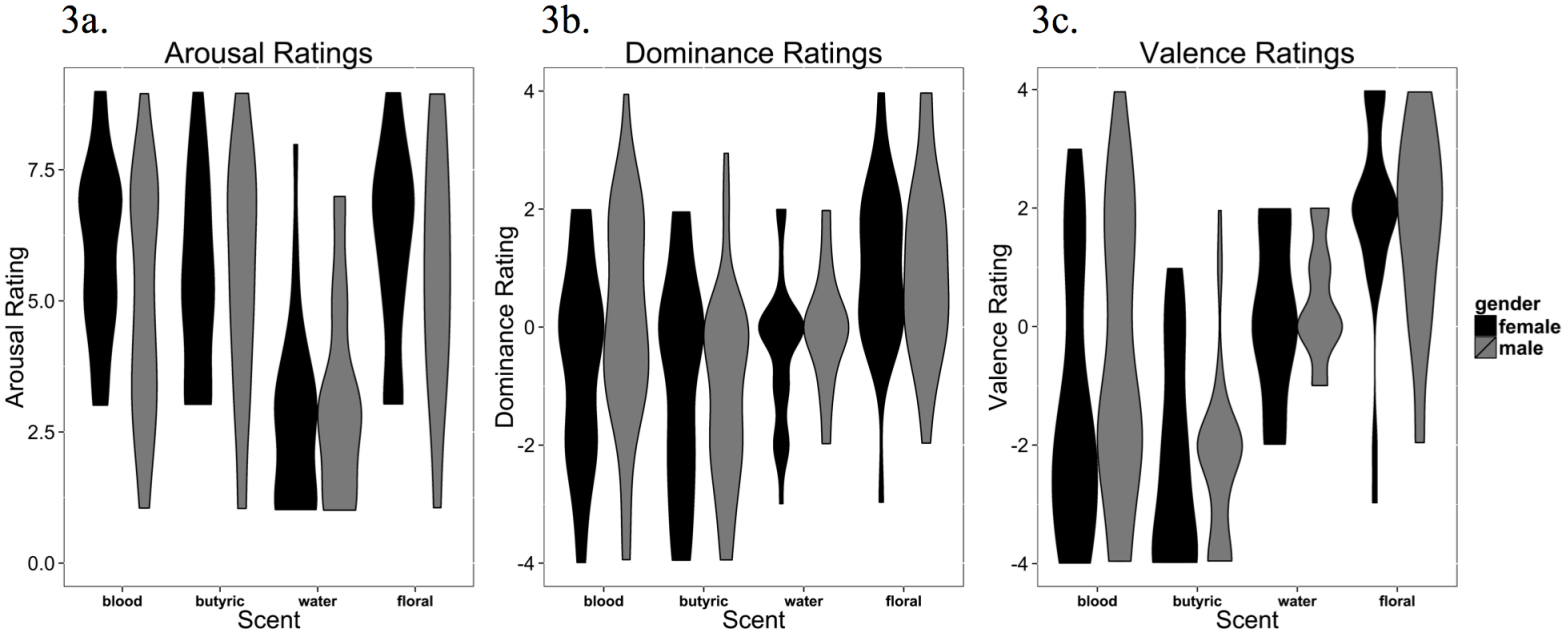
Probability density graph of SAM ratings. Simulated blood scent (‘blood’), butyric acid (‘butyric’), water, and floral odor (‘floral’), separated for gender. **a.** Arousal ratings. **b.** Dominance ratings. **c.** Valence ratings.

The distributions for all dilutions of simulated blood scent, butyric acid, water and floral odor are shown in Fig 3. Visual inspection of the data show consistently non-normal distributions irrespective of the odor tested. This was quantitatively supported by Shapiro-Wilkinson Tests, examining the samples of each of the 4 scents, split for gender, showed that the hypothesis of normality must be rejected (p < 0.05) for all but one category, this was floral scent valence/dominance/arousal in men (p > 0.05). Non-parametric statistics were therefore used to test overall differences between simulated blood scent and control odors, to see if simulated blood scent corresponded more to an unpleasant odor like butyric acid or pleasant odor like floral odor. Potential gender differences in ratings of simulated blood scent were also measured with non-parametric statistics.

#### Arousal

Friedman tests revealed an overall difference across the different scents (*X* ^*2*^(3) = 56.79, *p* < 0.0001), with Wilcoxon signed rank tests used as follow-up tests of simulated blood scent vs. control scents (α = 0.05/3 = 0.0167). These showed that simulated blood scent (*Mdn* = 5, [*Range*: 1 to 9]) was not significantly different from either butyric acid (*Mdn* = 5, [*Range*: 1 to 9], *Z = -* 0.67, *p* = 0.506, *r* = 0.10) or floral odor (*Mdn* = 6, [*Range*: 1 to 9], *Z = -* 0.85, *p* = 0.396, *r* = - 0.12), but was higher than water (*Mdn* = 3, [*Range*: 1 to 8], *Z* = 5.02, *p* < 0.0001, *r* = 0.73).

Mann-Whitney-U tests showed that women (*Mdn* = 6, [*Range*: 3 to 9]) rated simulated blood scent as significantly more arousing than did men (*Mdn* = 5, [*Range*: 1 to 9], *U* = 666, *p* = 0.035, *r* = 0.23). There was a correlation between simulated blood scent perception threshold and arousal in men (*r* = 0.36, *p* = 0.016), but not for women (*r* = - 0.25, *p* = 0.117).

#### Dominance

There was an overall difference between the different scents, as measured by a Friedman test (*X* ^*2*^(3) = 29.92, *p* < 0.0001), with Wilcoxon signed rank tests used as follow-up tests of simulated blood scent vs. control scents (α = 0.05/3 = 0.0167). These showed that simulated blood scent (*Mdn* = 0, [*Range*: -4 to 4]) was not significantly different from butyric acid (*Mdn* = -1, [*Range*: -4 to 3], *Z* = 2.10, *p* = 0.036, *r* = 0.31), or water (*Mdn* = 0, [*Range*: -3 to 2], *Z* = 0.49, *p* = 0.623, *r* = 0.07), but was significantly less dominant than floral odor (*Mdn* = 1, [*Range*: -3 to 4], *Z* = -3.94, *p* < 0.0001, *r* = - 0.57).

Mann-Whitney-U tests showed no significant difference in dominance ratings of simulated blood scent between women (*Mdn* = 0, [*Range*: -4 to 2]) and men (*Mdn* = 0, [*Range*: -4 to 4], *U* = 719.5, *p* = 0.106, *r* = - 0.18). There was no correlation between simulated blood perception threshold and dominance in either men (*r* = - 0.04, *p* = 0.780), or women (*r* = - 0.05, *p* = 0.743).

#### Valence

Friedman tests showed an overall difference across the different scents (*X* ^*2*^(3) = 76.10, *p* < 0.0001), with Wilcoxon signed rank tests used as follow-up tests of simulated blood scent vs. control scents (α = 0.05/3 = 0.0167). These showed that simulated blood scent (*Mdn* = -2, [*Range*: -4 to 3]) was significantly more positive than butyric acid (*Mdn* = -2, [*Range*: -4 to 2], *Z* = -2.90, *p* = 0.004, *r* = 0.42), but less positive than water (*Mdn* = 0, [*Range*: -2 to 2], *Z = -* 3.66, *p* < 0.0001, *r* = - 0.53), and floral odor (*Mdn* = 2, [*Range*: -2 to 4] *Z* = -5.22, *p* < 0.0001, *r* = - 0.76).

Mann-Whitney-U tests showed no significant difference in valence ratings of simulated blood scent between women (*Mdn* = -2, [*Range*: -4 to 3]) and men (*Mdn* = -1, [*Range*: -4 to 4], *U* = 713, *p* = 0.094, *r* = 0.18). There was no correlation between simulated blood scent perception threshold and valence in either men (*r* = - 0.29, *p* = 0.075), or women (*r* = - 0.11, *p* = 0.480).

**Fig 4:**
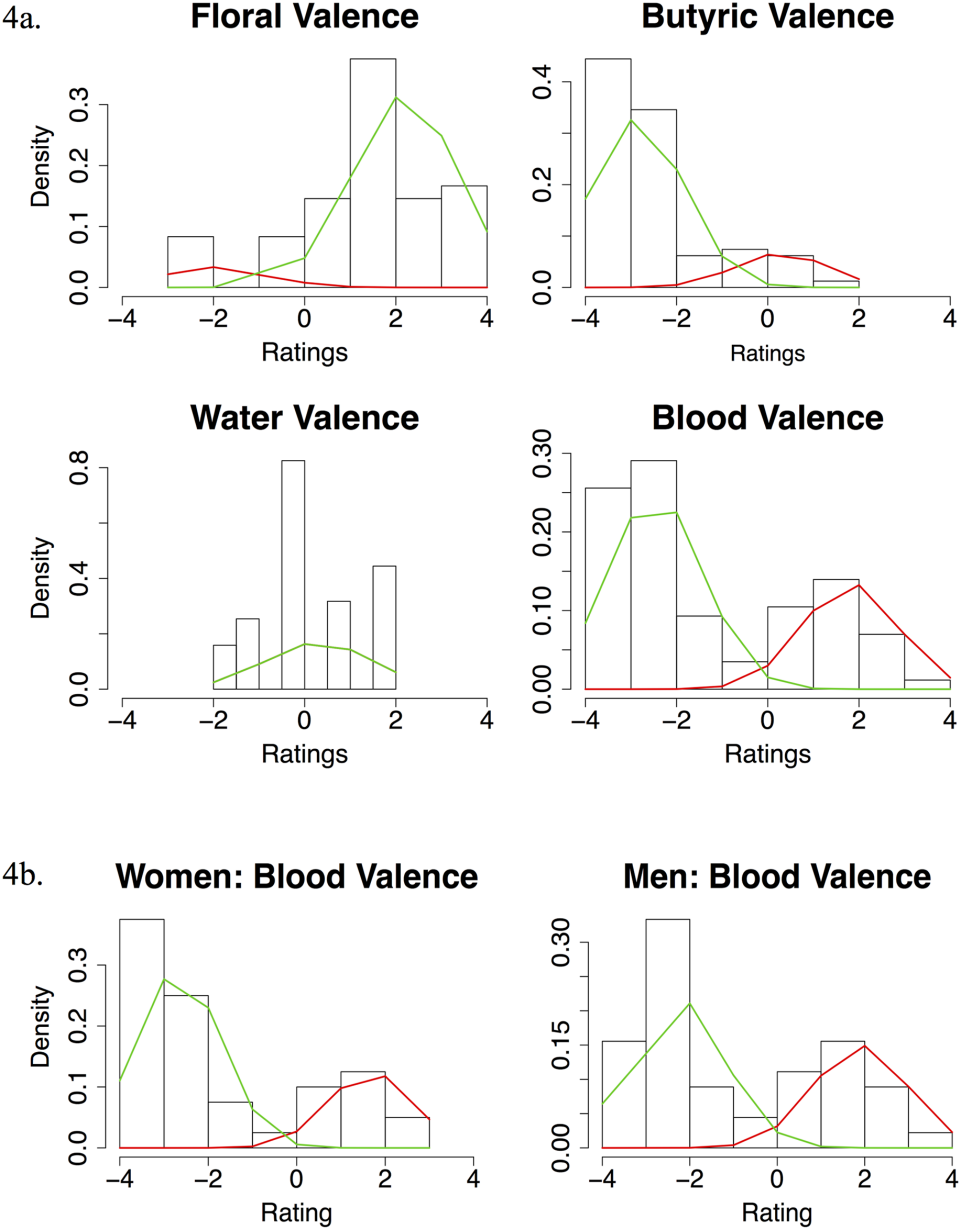
Bimodality tests. **a.** Histograms of valence ratings of 4 scents, with bimodal Gaussian distributions fitted. **b.** Bimodality test of valence ratings for simulated blood scent for men and women with bimodal Gaussian distributions fitted.

Visual inspection of the valence graphs suggested that simulated blood scent ratings are bimodal. To quantify this, we fitted three different models of Gaussian distributions to the data, with either 1, 2 or 3 peaks (given the *N* = 89 sample, we judged that any more would be over-fitting). We used the Akaike Information [49] to determine the best fit. Simulated blood scent, butyric acid and floral odor were all bimodal by these criteria, water was unimodal (see Fig 4a). The resultant density plots (created with [50]) showed butyric acid and floral odor heavily skewed to their predicted directions, with a small number of participants in the opposite directions. Simulated blood scent showed a more even split between people who liked it and disliked it. Repeating the model fitting for men and women separately replicated this bimodal split (see Fig 4b).

**Fig 5:**
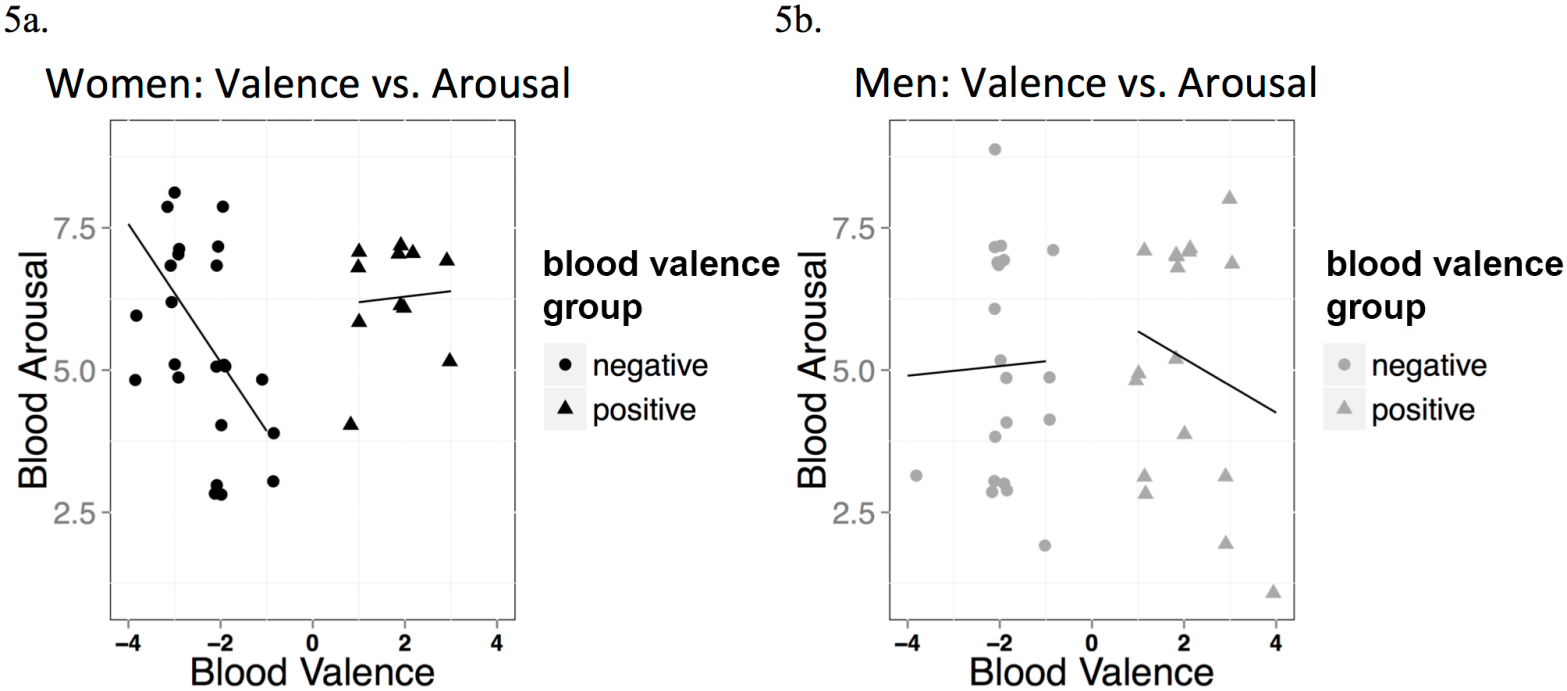
Valence vs. arousal scatterplots of simulated blood scent. Scatterplots for women (left) and men (right) showing relationship between scent valence ratings and arousal. Three zero scores for the whole sample, are removed from this graph.

Arousal and valence typically form a U-shaped distribution, with extremes of valence showing higher arousal [4]. This was partially present in the ratings of simulated blood scent valence and arousal in women. See Fig 5 for scatterplots. We split the valence ratings into positive and negative groups (eliminating the *N* = 3 who gave a response of ‘0’) and conducted non-parametric Spearman Rank correlations, which demonstrated that women who disliked the simulated blood scent were more likely to have a strong high arousal response (*r*_*s*_ = -0.62, *p* < 0.0001), whereas those who gave it a positive response did not show any correlation with arousal (*r*_*s*_ = -0.04, *p* = 0.906). Men showed no such connection between valence and arousal, either in a positive (*r*_*s*_ = -0.04, *p* = 0.869) or negative group (*r*_*s*_ = 0.03, *p* = 0.904).

### Fertility effects

#### Threshold

We tested the potential effect of women’s oral contraceptive use (non-OC/OC) and cycle stage (ovulatory phase/non-ovulatory phase) upon simulated blood scent and butyric acid threshold perception, creating 3 groups: Non-ovulatory phase, ovulatory phase and contraception. Although it is possible to split the contraception group further into cycle stages, and this has been shown to have effects [1], since different contraceptives have different effects on the cycle, it would be too complex to measure with this sample size. A Kruskall-Wallis non-parametric test evaluated the overall effect, showing no significant difference across all 3 possibilities for either simulated blood scent (*X* ^*2*^(2) = 2.25, *p* = 0.324) or butyric acid (*X* ^*2*^(2) = 1.56, *p* = 0.457).

#### Emotion Ratings

**Fig 6:**
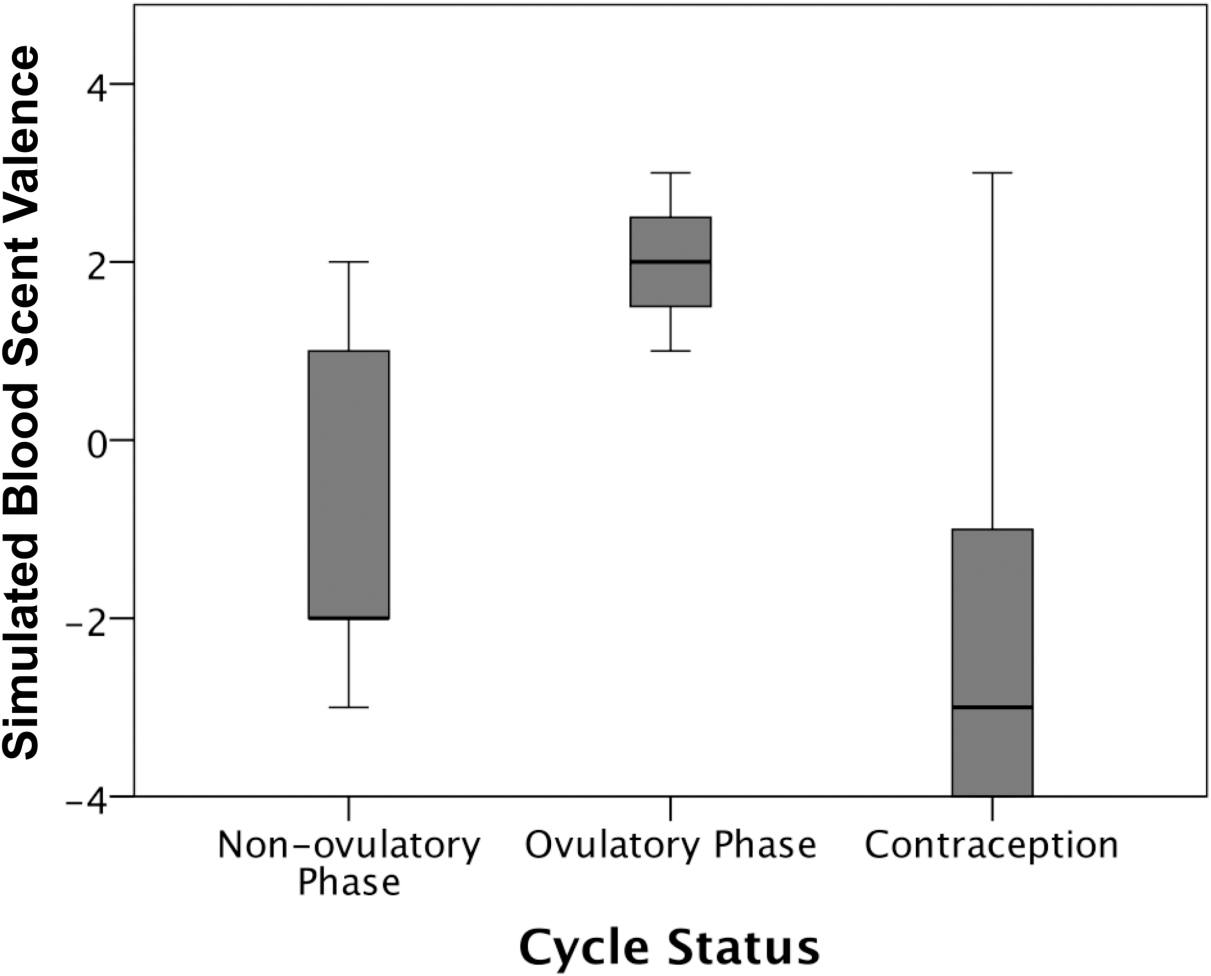
Women’s ratings of simulated blood scent. Box plot of women’s ratings of the valence of simulated blood scent according to whether they are taking contraception, and cycle stage (ovulation/non-ovulatory phase) of their menstrual cycle. Error bars represent +/-2 Standard deviations.

A Kruskall-Wallis test showed an overall effect of fertility and OC on simulated blood scent valence *X* ^*2*^(2) = 11.15, *p* = 0.004), which was not found for the other control scents (butyric acid: *X* ^*2*^(2) = 1.81, *p* = 0.404; water: *X* ^*2*^(2) = 0.51, *p* = 0.975; floral odor: *X* ^*2*^(2) = 0.32, *p* = 0.851), nor was it found for arousal responses to simulated blood scent (*X* ^*2*^(2) = 2.06, *p* = 0.358). Follow-up orthogonal contrasts on simulated blood scent tested effects of contraceptive use and ovulatory phase, finding that women who did not take contraceptives (*Mdn* = 1, [*Range*: -3 to 3]) liked the simulated scent of blood more than those who did take contraceptives (Mdn = -3, [*Range*: -4 to 3], *U* = 81.50, *p* = 0.004, *r* = 0.46). Amongst those women who did not take contraceptives, those that were also in their ovulatory phase (*Mdn* = 2, [*Range*: 1 to 3]) liked the simulated blood scent more than those in a non-ovulatory phase (*Mdn* = - 2, [*Range*: -3 to 2], *U* = 3.50, *p* = 0.015, *r* = 0.65). See Fig 6 for illustration.

### Meat-eating preferences

Mann-Whitney U tests showed that there was no significant difference in simulated blood scent valence ratings between people that consume meat (*Mdn* = 0, [*Range*: -4 to 3], *N* = 71), and vegans/vegetarians (*Mdn* = 0.5, [*Range*: -4 to 3], *N* = 18, *U* = 468.5, *p* = 0.23, *r* = - 0.13). Vegans/vegetarians (*Mdn* = 4, [*Range*: 1 to 9]) had lower arousal than the other groups (*Mdn* = 3, [*Range*: 1 to 7], *U* = 400, *p* = 0.046, *r* = - 0.22).

### Scent verbal association

We asked for associations with the simulated blood scent. Participants did not know what the scent was when they gave these associations. There was a lot of variance in these attributions, though certain ideas reoccurred e.g., cleaning mixture, hospitals, metallic, mushrooms, forest, stagnant water (see S1 Dataset for full list). The frequencies were too low to generate categories to calculate any conventional statistics.

## Discussion

The goal of this experiment was to obtain first psychophysical characteristics of people’s perception of and reaction to the simulated scent of fresh blood, particularly with respect to its potential relation to fundamental approach/withdrawal motivational states.

The perceptual threshold of simulated blood scent appears to be unimodal for both men and women. There are some scents, for example androstenone, for which different portions of the population have different genetically modulated receptor types, and thus different perceptual thresholds together with different valence ratings, [34]. The unimodality of the simulated blood scent is not unexpected, as in contrast to androstenone, the simulated scent of blood is a mixture, where one can expect a receptor for each component, with its own allelic variation. The holistic perception of blood scent would be of another order of complexity. These results have the practical implication for future studies that stimulus intensity will not have to be adjusted for two or more different populations. Women appear to be more sensitive to detecting simulated blood scent than men. Women are generally more sensitive to an array of human odors, such as breath, hand scents, andrenostone/ol, as well as non-human odors (see [1] for review). This study shows that simulated blood scent is not an exception. There are a variety of possible reasons for this generally higher sensitivity, most explanations favor fundamental biological differences, rather than differences of upbringing or hygiene [1]. Women’s everyday relationship with blood is different to that of men, via menstruation, and increased sensitivity might be a by-product of this. People can be sensitized to an array of different scents with repeated exposure, even if they are initially anosmic to them and this capability seems to be especially pronounced in women [51], especially for biological scents (e.g., androstenone [52]).

For the ratings of simulated blood scent, the most conspicuous result was the bimodality of valence ratings of blood scent, such that people either liked it or did not. This was the case for both genders. For women, the difference could be associated with differences in OC and the phase of their fertility, namely women who were not taking OC and who were in a point in their cycle corresponding to their fertile phase, showed a clearly more positive rating of the simulated blood scent. Previous research has shown that women vary significantly across the course of their cycle in their ratings of odors [1,26,53,54], indeed more intensive processing of scents has also been observed in ERPs [26]. It is not known why this is the case. It cannot be attributable to circulating ovarian hormones or gonadotrophic pituitary hormones. This is because women typically vary in their sensitivity and hedonic ratings of scents regardless of whether they are taking OC or not. Though research shows a generally more positive rating of scents in the time of ovulation, regardless of OC [1], data from this experiment cannot evaluate this, as the variety of different contraception methods have different effects upon the cycle.

Additionally, women found the simulated scent of blood more arousing than men, but this was only for those women who rated the scent as negative. Thus, although the literature suggests that women can be more sensitive to withdrawal cues [22], including olfactory cues [23,24], our findings limit this specifically to women taking OC. Women without OC rate it more positively, and this is further potentiated by the fertile phase of the menstrual cycle. Oral OC is known to have effects on the hypothalamic-pituitary axis [54], and women taking OC show less cortisol response to stress, suggesting that they might have less stress adaptability over the long term [55,56]. Additionally women taking OC have a greater emotional memory for emotional pictures, positive and negative [57]. One could interpret the results in this context as demonstrating that women taking OC are primed to interpret stimuli as alarm signals, and that blood scent in particular is interpreted as such.

We have no explanation for why men display a similar split in their hedonic ratings of blood scent. Through one rating it is as yet not possible to say whether it is a state or trait rating. Future experiments will clarify a possible contextual dependency [3]. People’s diverse reactions to the simulated scent of blood could reflect trait-like differences or they could reflect the fact that people can vary in their response to a stimulus according to their motivational state. An extreme example of the latter is in sexual response: Disgust and sexual arousal are two motivational states that are opposed to one another [58,59]. The former involves protection against contamination, the latter, fundamentally procreation. They often take the same stimuli: Mouths, genitalia, saliva, sexual effluvia, and body odors. The desire for sex is thus predicated on overcoming disgust. Studies have shown that sexual arousal diminishes disgust generally in both women [58] and men [59]. The responses to violent stimuli that mark reactive and appetitive aggression, show similar opposing qualities: The memory of an enemy soldier dying before one’s eyes can be a lifelong source of disgust and trauma for a veteran, or it can be an arousing and oft revisited memory [10]. Nell [7] describes the way chimpanzees hunting their prey switch from moment to moment from excitement at overwhelming the prey to fear as it defends itself. For a soldier, battle can be a mixture of terror and exhilaration, with ‘adrenaline’ becoming a drug [60]. Future studies need to examine the interplay of these two motivational systems. The bimodal distribution of blood scent could reflect blood’s suitability as a stimulus to test this. The fact that responses to blood scent varies across the menstrual cycle of women, peaking at a point of maximum fertility, could open up the possibility that it is somehow related to sexuality. Although the focus of this study was upon blood as a danger/appetitive signal, future studies could test the relation of people’s responses to blood scent against the spectrum of sexuality.

Another potentially interesting lifestyle relation to the scent of blood was whether people eat meat or not. Though this lifestyle is often chosen for ethical or health reasons, whether this position is reflected in a visceral response to blood scent is an open question. Though we did find a significantly lower arousal in people who were vegetarian or vegan, this is difficult to interpret, and a future study with a larger sample is required.

When asked to identify the blood scent, the student participants named a host of different associated scents: Cleaning materials, forest, mushrooms, hospitals, iron, swimming pools, two people mentioned blood, and one mentioned hemoglobin. In contrast, in a lecture, we described the rationale of our experiment, making it clear from the outset that we were running an experiment on the scent of blood, and gave the listeners a chance to smell the simulated scent. Many of these people confirmed that yes, this scent smells like e.g., ‘the slaughterhouse I grew up next to,’ or ‘of course, that is the coppery smell of menstrual blood’ etc. One could speculate that verbal cue, providing the context that this smells of blood, seems to alter conscious attributions. This is reinforced by laboratory experiments of other scents showing that people’s early neural encoding of the same scent will alter depending upon whether it is labeled in advance as something pleasant, unpleasant or neutral [61]. Visual cues could also have an effect, redness is itself a powerful environmental cue, and blood phobia fear responses are triggered by visual cues [13]. The underlying neurophysiological structures suggest that perception of visual and olfactory cues is holistically integrated [62]. Future studies will test this for blood scent. The hypothesis was that the response to simulated blood scent was evolutionarily conditioned and independent of life-experiences. However, one limitation of this experiment was in not assessing people’s previous experiences with blood, as this could have an effect on the ratings. More knowledge of the experiment participants’ experiences is therefore important for future experiments.

Future experiments can broaden the examination from the emotional aspects of motivation: Would it have a potentiating effect upon either appetitive or fearful responses, respectively, that is not found for other scents? Alternately, if the same people are measured over different time periods, will they show trait-like stability in their ratings of blood scent? The simulated blood scent mixture employed here provides a specific element of the scent of fresh blood that is convenient and safe for laboratory studies. This mixture is postulated to represent the scent of fresh blood as it mixes with the lipid peroxides on the skin [30]. However we do no yet know if this individual stimulus is comparable to the complexity of the real-life stimulus. Glindemann et al.’s [30] original assumption that the smell of blood in the natural world is ‘metallic’ was based upon a quantitative internet search, and analytical comparison of the reaction of blood on skin, with that of iron on skin. There is further empirical justification for the association of the scent of blood with ‘metallic’ qualities in nutrition science literature. The taste of blood-derived products is described as ‘metallic’, owing to its hemoglobin content [32,33], and the smell of oxidized porcine liver is specifically described as ‘metallic’ owing to the presence of 1-octen-3-one, one of the compounds of our study [31]. A direct comparison of the blood scent mixture with a real blood sample is important as a comparison of the stimulus, but this opens up many complications (stressed/non-stressed, human/non-human, fresh/oxidized, male/female, old/young), which only a large number of future studies could fully address. Blood has many other components that impact behavior, in particular, a dying prey animal’s blood contains stress hormones, which could also conceivably excite a predator or frighten a conspecific [20,21]. On the other hand, predatory animals have been shown to react to an individual volatile component of blood itself (trans-4,5-epoxy-(E)-2-decenal) in the same appetitive way as with real blood [6]. At a theoretical level, identifying a biologically important environmental scent cue from one of its components could provide an advantage in sensitivity. But, configural perception of multiple elements of a scent is advantageous in the complex changing natural environment, moreover, if it were dependent only upon only one receptor, there is the possibility of a specific anosmia, which could be disastrous for an animal [63]. Blood is a complex stimulus, and this experiment shows complex responses in people to a major distinctive component of this.

In sum, this experiment examines the novel question concerning the effects of the scent of blood on humans. This preliminary data affirm the idea that its olfactory perception represents an important chemosensory signal requiring further investigation.

## Supporting Information

S1 Dataset Raw Dataset of experiment. (XLS)

S1 Text Odour Stimulus Parameters. Description of creation of odor stimuli, with all concentrations included.
(DOCX)
