# Supplementary material for: The Scent of Blood: A Driver of Human Behavior?
Source: PLoS One. 2015 Sep 23;10(9):e0137777. doi: 10.1371/journal.pone.0137777 (PMC4580480; doi:10.1371/journal.pone.0137777)
Supplement: S1 Text — Description of creation of odor stimuli, with all concentrations included. (DOCX) [file pone.0137777.s002.docx]

# Supporting Information 2: Odour Stimulus Parameters

Based on the previous literature that suggested that a mixture of hexanal, hepatanal, octanal, nonanal, decanal and 1-octen-3-one could create a similar scent as the one of “blood” (Glindemann et al., Angew. Chem. Int. Ed. 2006, 45, 7006 –7009). This mixture was recreated using compounds listed in Table 1. Table 2 demonstrates some basic calculations derived from making dilutions of individual compounds in the mixture as well as the mixture itself and the amount that could be generated when applying 1 µl of the dilutions generated. Similar dilutions and calculations were carried out for the two positive controls butyric acid and rose oil (phenethyl ethanol). All calculations in Table 2 demonstrate that even when applying the most concentrated dilutions, the actual levels of the compounds employed are extremely low.

Table 1: Compounds

|  | Substance (10nl) | CAS # | Density  [g/ml] | MW  [g/mol] |
| --- | --- | --- | --- | --- |
| 1 | Hexanal | 66-25-1 | 0.802 | 100.16 |
| 2 | Heptanal | 111-71-7 | 0.809 | 114.2 |
| 3 | Octanal | 124-13-0 | 0.828 | 128.2 |
| 4 | Nonanal | 124-19-6 | 0.827 | 142.24 |
| 5 | Decanal | 112-31-2 | 0.830 | 156.27 |
| 6 | 1-octen-3-one | 4312-99-6 | 0.826 | 126.19 |
| 7 | Butyric Acid | 107-92-6 | 0.959 | 88.11 |
| 8 | Phenethyl ethanol (Rose oil) | 60-12-8 | 1.0202 | 122.16 |

Table 2: Dilution and mixture calculations

|  | Substance (10nl) | Solution of 10 nl concentrate in 1ml EtOH  [µg/ml] | 1 µl Solution: Concentration at 4 Liter^A^ air throughput, 100% Volatilisation  [µg/m3] | nMol/m^3^ |
| --- | --- | --- | --- | --- |
| 1 | Hexanal | 8,02 | 2,005 | 20,01 |
| 2 | Heptanal | 8,09 | 2,025 | 17,73 |
| 3 | Octanal | 8,28 | 2,07 | 16,15 |
| 4 | Nonanal | 8,27 | 2,07 | 14,55 |
| 5 | Decanal | 8,30 | 2,075 | 13,28 |
| 6 | 1-octen-3-one | 8,26 | 2,6 | 20,60 |
| 7 | Mix of 1-6 | Σ 10 nl Conc. of 1-6 in 1 ml EtOHSolution | - | Σ Pos 1-6 = 302,32 |
| 8 | Butyric Acid | 9,59 | 2,3975 | 27,21 |
| 9 | Phenethyl ethanol (Rose oil) | 10,202 | 2,5505 | 20,89 |

^A^ Volunteer exposure scenario is 400 ml air per minute for 10 minutes = 4 liters in total.

The “synthetic blood mixture” was generated via a 1: 10 dilution of the “Mix 1-6” in Table 2 in water (Table 3). The corresponding final concentration of each compound within this synthetic blood mixture was also calculated and is also shown in Table 3. From this “synthetic blood mixture” a serial dilution in water using half-logarithmic steps was generated and the ensuing individual concentrations of each compound in that mixture per the respective dilution step calculated (Table 4). A similar procedure was employed for the two positive controls rose oil and butyric acid with the exception that at the outset 15 ml of the undiluted compounds were employed as the starter concentration. Analogous to the dilution series of the “synthetic blood mixture” serial dilutions of rose oil and butyric acid in water was generated using half-logarithmic steps and the ensuing individual concentrations of each compound in that respective dilution step calculated (Table 5).

Table 3:Original synthetic blood mixture employed

| Substance (10nl) | Solution of 10 nl concentrate in 1ml EtOH  [µg/ml] | Blood Mixture  1:10 dilution of EtOH solution in 1 ml H_2_O |
| --- | --- | --- |
| Mix of hexanal, hepatanal, octanal, nonanal, decanal and 1-octen-3-one | Σ 10 nl conc. of hexanal, hepatanal, octanal, nonanal, decanal and 1-octen-3-one in 1 ml EtOH | 0,802 µg Hexanal  0,809 µg Heptanal  0,828 µg Octanal  0,827 µg Nonanal  0,830 µg Decanal  0,826 µg 1- octen-3-one |

Table 4: Dilutions of the blood mix employed and the corresponding concentrations of each individual component of the blood mix within 1 ml at the respective dilution

| Blood Mix ml  ad 15 ml H_2_O Half-log dilution series | Hexanal  [ng/ml mix] | Heptanal  [ng/ml mix] | Octanal  [ng/ml mix] | Nonanal  [ng/ml mix] | Decanal  [ng/ml mix] | 1-octen-3-one  [ng/ml mix] |
| --- | --- | --- | --- | --- | --- | --- |
| 15 | 401 | 404,5 | 414 | 413,5 | 415 | 413 |
| 4,74341649 | 126,80733 | 127,9141 | 130,9182 | 130,7601 | 131,234 | 130,6021 |
| 1,5 | 40,1 | 40,45 | 41,4 | 41,35 | 41,5 | 41,3 |
| 0,474341649 | 12,680733 | 12,79141 | 13,09182 | 13,07601 | 13,1234 | 13,06021 |
| 0,15 | 4,01 | 4,045 | 4,14 | 4,135 | 4,15 | 4,13 |
| 0,047434165 | 1,2680733 | 1,279141 | 1,309182 | 1,307601 | 1,31234 | 1,306021 |
| 0,015 | 0,401 | 0,4045 | 0,414 | 0,4135 | 0,415 | 0,413 |
| 0,004743416 | 0,1268073 | 0,127914 | 0,130918 | 0,130760 | 0,13123 | 0,130602 |
| 0,0015 | 0,0401 | 0,04045 | 0,0414 | 0,04135 | 0,0415 | 0,0413 |
| 0,000474342 | 0,0126807 | 0,012791 | 0,013091 | 0,013076 | 0,01312 | 0,013060 |
| 0,00015 | 0,00401 | 0,004045 | 0,00414 | 0,004135 | 0,00415 | 0,00413 |
| 4,74342E-05 | 0,0012680 | 0,001279 | 0,001309 | 0,001307 | 0,00131 | 0,001306 |
| 0,000015 | 0,000401 | 0,000404 | 0,000414 | 0,000413 | 0,00041 | 0,000413 |
| 4,74342E-06 | 0,0001268 | 0,000127 | 0,000130 | 0,000130 | 0,00013 | 0,000131 |
| 0,0000015 | 0,0000401 | 0,000040 | 0,000041 | 0,000041 | 0,00004 | 0,000041 |
| 4,74342E-07 | 1,268E-05 | 1,27E-05 | 1,30E-05 | 1,30E-05 | 1,3E-05 | 1,31E-05 |

Table 5: Dilutions of the positive controls, rose oil (Phenethyl ethanol) and butyric acid and the corresponding concentrations of each individual component within 1 ml at the respective dilution

| Roseoil or Butyric Acid ml  ad 15 ml H_2_O Half-log dilution series | Rose Oil  [µg/ml mix] | Butyric Acid  [ng/ml mix] |
| --- | --- | --- |
| 15 | 510,1 | 479,5 |
| 4,74341649 | 161,30778 | 151,6312 |
| 1,5 | 51,01 | 47,95 |
| 0,474341649 | 16,130778 | 15,16312 |
| 0,15 | 5,101 | 4,795 |
| 0,047434165 | 1,6130778 | 1,516312 |
| 0,015 | 0,5101 | 0,4795 |
| 0,004743416 | 0,1613078 | 0,151631 |
| 0,0015 | 0,05101 | 0,04795 |
| 0,000474342 | 0,0161308 | 0,015163 |
| 0,00015 | 0,005101 | 0,004795 |
| 4,74342E-05 | 0,0016131 | 0,001516 |
| 0,000015 | 0,0005101 | 0,000480 |
| 4,74342E-06 | 0,0001613 | 0,000151631 |
| 0,0000015 | 0,0000510 | 0,000048 |
| 4,74342E-07 | 1,613E-05 | 1,516E-05 |
